# Acute Exercise in Hypobaric Hypoxia Attenuates Endothelial Shedding in Subjects Unacclimatized to High Altitudes

**DOI:** 10.3389/fphys.2019.01632

**Published:** 2020-02-04

**Authors:** Julia M. Kröpfl, Tobias Kammerer, Valentina Faihs, Hans-Jürgen Gruber, Jan Stutz, Markus Rehm, Ingeborg Stelzer, Simon T. Schäfer, Christina M. Spengler

**Affiliations:** ^1^Exercise Physiology Lab, Institute of Human Movement Sciences and Sport, ETH Zürich, Zurich, Switzerland; ^2^Department of Anaesthesiology, Ludwig Maximilian University of Munich, Munich, Germany; ^3^Walter Brendel Centre of Experimental Medicine, Ludwig Maximilian University of Munich, Munich, Germany; ^4^Institute of Anesthesiology, Heart and Diabetes Center NRW, Ruhr-University Bochum, Bad Oeynhausen, Germany; ^5^Clinical Institute of Medical and Chemical Laboratory Diagnostics, Medical University of Graz, Graz, Austria; ^6^Institute of Medical and Chemical Laboratory Diagnostics, LKH Hochsteiermark, Leoben, Austria; ^7^Zurich Center for Integrative Human Physiology (ZIHP), University of Zurich, Zurich, Switzerland

**Keywords:** high altitude, endothelial shedding, hematopoietic progenitor cell, matrix remodeling, unacclimatized

## Abstract

Travel of unacclimatized subjects to a high altitude has been growing in popularity. Changes in endothelial shedding [circulating endothelial cells (ECs)] and hematopoietic stem and progenitor cells (CPCs) during physical exercise in hypobaric hypoxia, however, are not well understood. We investigated the change in ECs and CPCs when exposed to high altitude, after acute exercise therein, and after an overnight stay in hypobaric hypoxia in 11 healthy unacclimatized subjects. Blood withdrawal was done at baseline (520 m a.s.l.; baseline), after passive ascent to 3,883 m a.s.l. (arrival), after acute physical exercise (±400 m, postexercise) and after an overnight stay at 3,883 m a.s.l. (24 h). Mature blood cells, ECs, and CPCs were assessed by a hematology analyzer and flow cytometry, respectively. The presence of matrix metalloproteinases (MMPs), their activity, and hematopoietic cytokines were assessed in serum and plasma. EC and CPC concentrations significantly decreased after exercise (*p* = 0.019, *p* = 0.007, respectively). CPCs remained low until the next morning (24 h, *p* = 0.002), while EC concentrations returned back to baseline. MMP-9 decreased at arrival (*p* = 0.021), stayed low postexercise (*p* = 0.033), and returned to baseline at 24 h (*p* = 0.035 to postexercise). MMP-activity did not change throughout the study. Circulating MMP-9 concentrations, but not MMP-activity, were associated with EC concentrations (*r*_rm_ = 0.48, *p* = 0.010). CPC concentrations were not linked to hematopoietic cytokines. Acute exercise at high altitude attenuated endothelial shedding, but did not enhance regenerative CPCs. Results were not linked to endothelial matrix remodeling or CPC mobilization. These results provide information to better understand the endothelium and immature immune system during an active, short-term sojourn at high altitude.

## Introduction

It has become more popular for the general population to enjoy travel to and exercise at high altitude. Such short-term travel and expeditions can be personally rewarding, but does come with the risk of not being able to cope with the challenging environment, especially seen in unacclimatized subjects (Beidleman et al., 2018). Exposure to hypobaric hypoxia at rest comes with an increase in heart rate (HR) and ventilation, a decrease in oxygen saturation, and an activation of angiogenesis and the endothelium (Luks, 2015) by, e.g., increased shedding of endothelial cells (ECs) from the vascular walls (Rabelink et al., 2010). The effect of acute exercise under hypoxic conditions on EC number, however, is controversial. Although studies have found a decrease in endothelial shedding after a 12-day trek at high altitude (Mancuso et al., 2008), acute physical exercise for 30 min in normobaric hypoxia showed elevated EC numbers (Tsai et al., 2016). Since endothelial shedding has been connected to adverse cardiovascular events including stroke (Bartsch and Gibbs, 2007; Deng et al., 2017), EC changes by acute exercise at high altitude, possibly being exercise-dose dependent by exercise-increased cardiac output and shear stress, should be explored.

For the prevention of adverse cardiovascular events, the number of circulating hematopoietic and endothelial stem and progenitor cells (CPCs) is also of importance. In case of hypoxia-induced endothelial dysfunction, CPCs help regenerate the endothelium (Rabelink et al., 2010). CPC response to acute hypoxia alone and under exercise conditions is contradictory. Studies found both an acute reduction (Colombo et al., 2012) and increase (Ciulla et al., 2007) in CPC number within 30–240 min of normobaric hypoxia exposure. Acute incremental cycling exercise in normobaric hypoxia elevated CPC number shortly after exercise cessation (Kroepfl et al., 2012), as did an active sojourn at low altitudes (Theiss et al., 2008), while a 12-day trek at high altitude showed a CPC reduction in peripheral blood (Mancuso et al., 2008). Thus, the questions arise if CPCs decrease or increase at high altitude and if acute physical exercise under these extreme conditions could counteract the hypoxia-induced effect. Exercise-induced CPC change at high altitude could also be exercise-intensity dependent (Agha et al., 2017).

Different circulating proteins have been shown to modulate exercise-induced EC and CPC concentrations such as enzymes related to endothelial matrix remodeling [matrix metalloproteinases, (MMPs); tissue inhibitor of MMPs (TIMP)] (Ross et al., 2014) and hematopoietic cytokines [stromal cell-derived factor (SDF-1α); interleukin-3 (IL-3)] (Emmons et al., 2016). In detail, MMP-2 and MMP-9 are gelatinases, proteolytic enzymes secreted from many cell types such as ECs, monocytes, and macrophages (Newby, 2008) and are responsible for remodeling and degrading the basement membrane and endothelium upon leukocyte transendothelial migration via VCAM-1 (Cook-Mills, 2006). They are regulated by one of their inhibitors (TIMP-1). Although the circulating concentrations of MMPs might be important information for protein presence (Cui et al., 2017), regarding endothelial matrix remodeling, their ability to degrade several types of collagen (MMP-activity) is even more meaningful. The presence of MMP protein is not necessarily indicative of MMP enzymatic activity, especially when samples are taken from peripheral blood and not at the tissue level (Lo Presti et al., 2017). SDF-1α is a potent chemoattractant for CPCs and regulates cell cycle status, cell adhesion, and survival (Alvarez et al., 2013). In addition, migration of bone marrow cells toward SDF-1α is enhanced by IL-3 preconditioning and upregulation of chemokine receptor-4 expression on the cells’ surface (Barhanpurkar-Naik et al., 2017).

The aim of the present study was therefore (1) to investigate the changes of EC and CPC concentrations after an ascent to high altitude (3,883 m a.s.l.), an acute bout of physical exercise, and an overnight stay; (2) to investigate if cell change during the acute exercise bout at high altitude would be exercise-dose dependent; and (3) to explain measured cell concentrations by the influence of exercise-induced cell modulators. Our hypotheses are as follows. Acute hypobaric hypoxia would increase EC and suppress CPC concentrations. Both EC and CPC numbers would increase after 2 h of acute physical exercise in hypobaric hypoxia. Cell concentrations would be back to pre-exercise levels after one night at altitude. Absolute changes of both cell types would depend on relative exercise dose. EC concentrations would not be associated with MMP-9 or MMP-2 protein concentrations, but only with MMP-activity, and CPC concentrations would be related to SDF-1α and IL-3 plasma concentrations in circulation.

## Materials and Methods

### Ethics Statement

Written informed consent was obtained for all subjects. The study conformed to the standards set by the Declaration of Helsinki, except for registration in a database, and the procedures were approved by the local Ethics Committee of the University of Munich (project no. 350-16).

### Subject Characteristics and Study Design

Eleven healthy subjects (age, 36.4 (7.0) years; BMI, 22.7 (2.0) kg m^–2^) were investigated. This study was part of a larger research expedition conducted in October 2016 (Kammerer et al., 2018). However, the primary research questions addressed in the current paper are novel and are exclusively dealt within this study alone with no overlap between this investigation and others completed on the research expedition. Exclusion criteria were infection, immunological disorders, pregnancy, any kind of preexisting cardiopulmonary disease, and exposition to altitudes higher than 2,000 m a.s.l. within 2 months before the study.

Blood withdrawal was done at baseline (Munich, Germany, 520 m a.s.l.; baseline) on day 1 at 8 AM, on day 2 after spending one night at 1,620 m a.s.l. (Zermatt, Switzerland) ascending by cable car to 3,883 m a.s.l. (Glacier Paradise; arrival) at 10 AM, after 2 h of physical exercise (400 m descent and ascent, postexercise) and on day 3 after an overnight stay at 3,883 m a.s.l. (24 h) at 8 AM. During exercise, HR was recorded via chest belt and wrist watch (Polar S610i, Polar Electro, Kempele, Finland), and activity counts were measured by an activity monitor (Actiwatch, Cambridge Neurotechnology Ltd, Cambridgeshire, United Kingdom). Postexercise measurements were performed in an expedition tent (Keron 4 GT, Hilleberg AB, Frösön, Sweden) on the glacier at 3,883 m a.s.l.

For the 2 h of acute physical exercise, subjects were asked to walk independently and as fast as possible in view of the given changes in distance and altitude.

During the study, one subject met the exclusion criteria and was excluded from further analyses. For three other subjects, HR data are missing, and for one additional subject, activity monitoring was not possible due to technical problems.

### Blood Cell Count and Serum Analysis

At each timepoint, 20 ml of peripheral blood was collected in lithium heparin tubes, and blood cell counts were measured using a hematology analyzer (ADVIA 2120i, Siemens, Zurich, Switzerland) (Kim et al., 2012). White blood cell counts were read from the peroxidase channel. If necessary, samples were diluted (1:3, 1:8). Platelet counts are not displayed in the results section, since lithium-heparin anticoagulated blood is more prone to platelet clumping and might bias outcome (Nelles and Chandler, 2014).

Additional blood was collected in 9 ml serum and ethylenediaminetetraacetic acid tubes (Sarstedt, Nümbrecht, Germany). Tubes were centrifuged according to the manufacturer’s instructions and immediately frozen (−80°C) until analysis. Parameters of endothelial matrix remodeling (MMP-2 and -9; TIMP-1) were determined by ELISAs (R&D Systems Europe, United Kingdom). MMP activity was analyzed using Fluorogenic Peptide Substrate (R&D Systems Europe, United Kingdom) (Bernecker et al., 2011). Parameters of hematopoietic stem cell mobilization (SDF-1α; IL-3) were measured by Procarta multiplex cytokine kit (Affymetrix, Santa Clara, CA, United States) (Schafer et al., 2016). Inter- and intra-assay CVs for ELISAs were below 15%.

### Cell Analysis

Mononuclear cells were isolated by density gradient centrifugation (Histopaque^®^-1077, Sigma-Aldrich, Buchs, Switzerland, Cat#10771), within 6 h after blood withdrawal. Briefly, 10^6^ mononuclear cells in phosphate-buffered saline including fetal bovine serum (2%) and ethylenediaminetetraacetic acid (0.4%) were labeled by antibodies CD34-phycoerythrin (PE, clone 581, BD Biosciences, Allschwil, Switzerland, Cat#555822), CD45-fluorescein-isothiocynate (FITC, clone HI30, Thermofisher, Schlieren, Switzerland, Cat#MHCD4501), and CD31-allophycocyanin-Cy7 (APC-Cy7, clone WM59, Lucerna-Chem AG, Lucerne, Switzerland, Cat#303120) and incubated for 30 min on ice in the dark. After incubation, samples were washed and further incubated with a live/dead stain (LIVE/DEAD^TM^ Fixable Aqua Dead Cell Stain Kit, Thermofisher Scientific, Zurich, Switzerland, Cat#L34965) for 15 min at room temperature in the dark. After incubation, samples were washed and finally fixated with 4% paraformaldehyde in phosphate-buffered saline (Thermo Fisher Scientific, ON, Canada, Cat#AAJ19943K2). Fluorescent minus one samples were used as negative controls. Three-color analysis was performed immediately after staining with compensated fluorescent parameters (BD^TM^ CompBead, BD Biosciences, Allschwil, Switzerland, Cat#L34965). The acquisition gate was established based on forward and side scatter characteristics including lymphocytes, but excluding granulocytes, monocytes, and debris. Cell subgroups, namely, CPCs (CD34 + /CD45dim) and mature ECs (CD34dim/CD45-/CD31+) (Duda et al., 2007; Bellows et al., 2011), were counted by a FACSCanto2 flow cytometer (equipment of the flow cytometry facility, University of Zurich, Switzerland) using a FACSDiva software (BD Biosciences, Allschwil, Switzerland) and a separate analysis tool (FlowJo, LLC, Oregon, United States). In our laboratory, inter- and intra-assay CVs for both CPC and EC counting by flow cytometry are below 10%. Estimates of the absolute numbers of total CPCs and ECs were given as cells/microliter calculated by multiplying the percentage of each cell subset of the lymphocyte gate by the absolute number of lymphocytes in peripheral blood measured by a standard hematology analyzer (Kropfl et al., 2019). Since hemoglobin concentration did not change throughout the study duration, estimated cell results did not have to be corrected for a shift in hemoconcentration (Matomaki et al., 2018).

### Statistics

Data are presented as individual values or mean with standard deviation (SD). HR for the full exercise bout is presented as time-weighted average, which was calculated by

$$\frac{duration_{down}{\;}^{*}\;\%HR_{pred,\;down}}{duration_{total}}+\frac{duration_{up}{\;}^{*}\;\%HR_{pred,\;up}}{duration_{total}}$$

Variables were tested for normal distribution by the Kolmogorov–Smirnov test. *A priori* power analysis revealed a sample size of *n* = 8 to detect a significant exercise-induced rise of CPCs 10 min after exercise cessation under hypoxic conditions (Kroepfl et al., 2012). A repeated-measures ANOVA including Bonferroni *post hoc* corrections was used to evaluate changes of progenitor and mature blood cells as well as serum and plasma parameters over time. Extreme values were excluded from analysis if they were >3 times the interquartile range. Pearson’s product-moment (coefficient *r*) or repeated-measures correlation (coefficient *r*_*rm*_) analyses were used to determine the relationship between variables. Unlike simple correlation, repeated-measures correlation analysis does not violate the assumption of independence of observations and tends to have much greater statistical power in the case of a repeated-measures design. The slopes that are displayed identify the best fit for all intraindividual associations (Bakdash and Marusich, 2017). Calculations were done with R3.3.0 using the available package “rmcorr.” In addition, G^∗^Power3.1.7, IBM SPSS Statistics25, and GraphPad Prism8 were used for analyses and visualizations. A *p* < 0.05 was considered as significant.

## Results

### Exercise Dose

During descent, subjects walked for 48.9 (10.2) min at 70.4 (9.2)% of predicted HR_*max*_ (Tanaka et al., 2001); during the immediately following ascent, they walked for 66.4 (13.5) min at 86.7 (6.3)% of predicted HR_max_. During the full exercise bout, subjects walked for 115.3 (14.8) min at 79.2 (7.1)% of predicted HR_max_. Activity counts were 54,473 (11,267.9) for the descent, 67,522.6 (9,891.3) during the ascent, and 121,995.6 (14,395.6) during the full exercise bout.

### Circulating Endothelial Cell Number (Endothelial Shedding)

**FIGURE 1:**
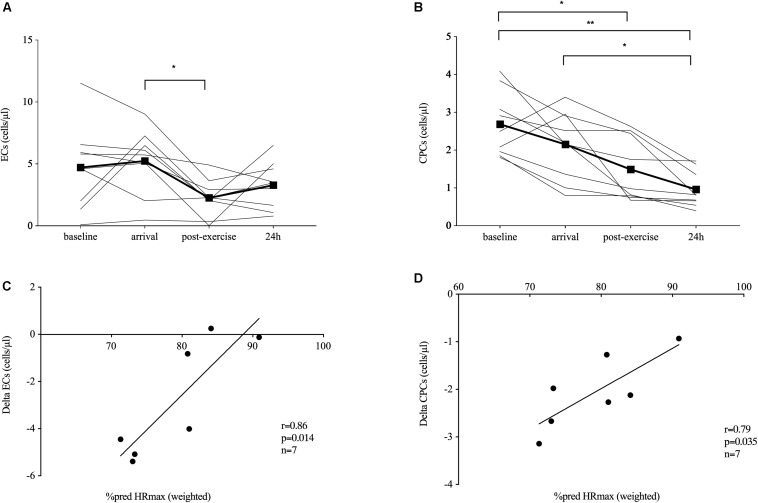
Endothelial shedding and immature immune cell response. Endothelial shedding [circulating endothelial cells, ECs, *n* = 9 **(A)**] and hematopoietic stem and progenitor cells [CPCs, *n* = 9, **(B)**] at baseline (520 m a.s.l.) and in response to exposure of hypobaric hypoxia after passive ascent to an altitude of 3,883 m a.s.l. (arrival), after 2 h of acute hiking exercise (± 400 m elevation gain, postexercise) and an overnight stay (24 h) at 3,883 m a.s.l. Absolute EC **(C)** and CPC **(D)** changes during the acute exercise bout were significantly related to the relative exercise dose given by the time-weighted average heart rate (HR) as percent predicted of HR_max_. Significant differences are indicated by *p ≤ 0.05, **p < 0.01 and were assessed by repeated-measures ANOVA with Bonferroni *post hoc* comparisons. Parameter associations were analyzed by Pearson’s correlation analysis.

Spending one night at 1,620 m a.s.l. and passively ascending to 3,883 m a.s.l. (baseline–arrival) did not significantly change EC numbers. After 2 h of hiking in hypobaric hypoxia, EC concentrations significantly decreased (−59%, *p* = 0.026) and returned back to concentrations measured at arrival and baseline at 24 h (Figure 1A).

### Circulating Hematopoietic Stem and Progenitor Cell Number

Spending one night at 1,620 m a.s.l. and passively ascending to 3,883 m a.s.l. (baseline–arrival) did not significantly change CPC numbers. After 2 h of hiking, CPCs significantly decreased (−30%, *p* = 0.011 compared to baseline), and CPC concentrations remained low until 24 h compared to baseline (−64%, *p* = 0.001) and arrival (−55%, *p* = 0.014; Figure 1B).

### Cell and Exercise Dose Correlations

Absolute EC and CPC change during the acute exercise bout was significantly related to HR as percent of predicted HR_max_ (*r* = 0.86, *p* = 0.014; and *r* = 0.79, *p* = 0.035, respectively; *n* = 7, Figures 1C,D). Analyzing the descent and ascent separately, only HR as percent of predicted HR_max_ during descent correlated with absolute EC change (*r* = 0.92, *p* = 0.003, *n* = 7, data not shown).

### Parameters of Endothelial Matrix Remodeling

**FIGURE 2:**
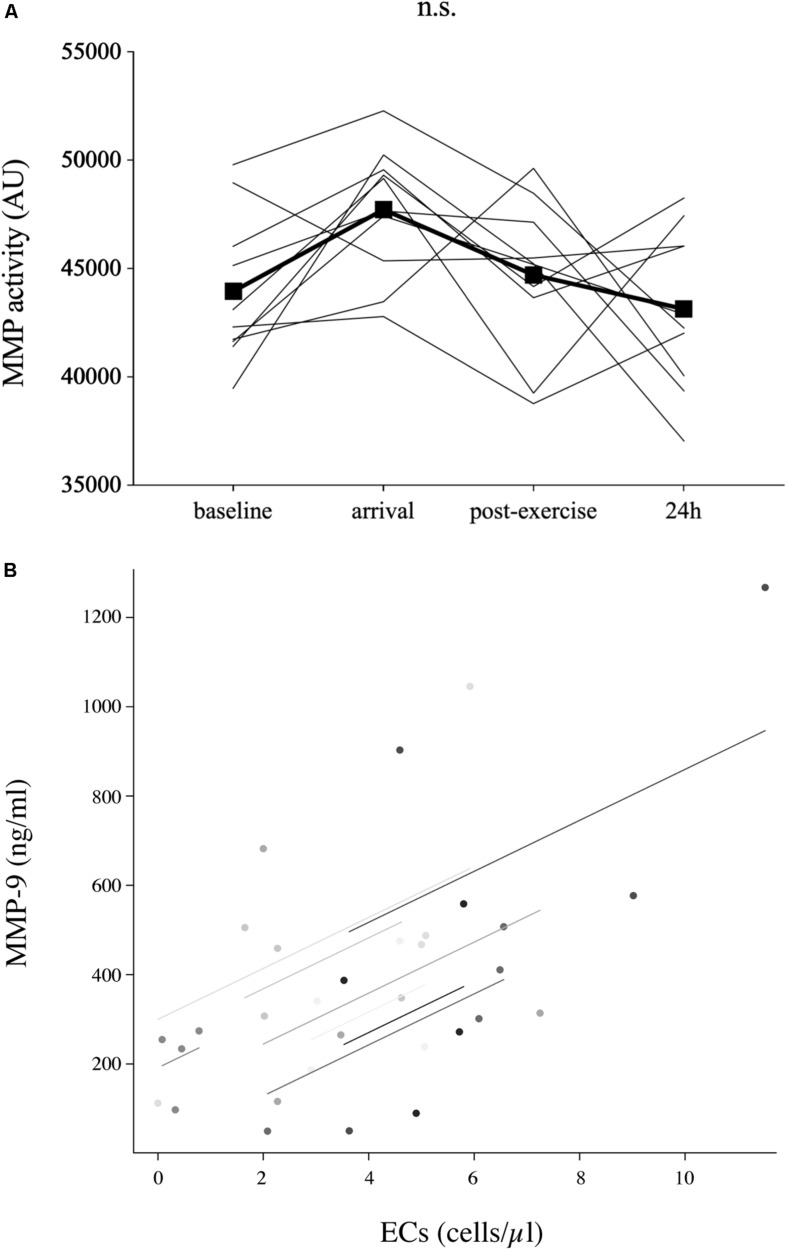
Parameters of endothelial matrix remodeling. The activity of matrix metalloproteinase (MMP) (*n* = 10) at baseline (520 m a.s.l.) and in response to exposure of hypobaric hypoxia after passive ascent to an altitude of 3,883 m a.s.l. (arrival), after 2 h of acute hiking exercise (±400 m elevation gain, postexercise) and an overnight stay (24 h) at 3,883 m a.s.l **(A)**. Only circulating MMP-9 concentrations, not MMP activity, were associated with EC concentrations [*r*_rm_ = 0.48, *p* = 0.010, *n* = 9 **(B)**]. Analysis was done by repeated-measures ANOVA with Bonferroni *post hoc* comparisons. The relationship between parameters was analyzed by repeated-measures correlation. n.s., non-significant.

Circulating MMP-9 values (Table 1) decreased below baseline at arrival (−46%, *p* = 0.021) and stayed low in 9 out of 10 subjects postexercise (−76%, *p* = 0.033, compared to baseline). At 24 h, MMP-9 concentrations were back to baseline (+188%, *p* = 0.035 to postexercise). MMP-2 concentrations (Table 1) were significantly elevated at arrival (+17%, *p* = 0.001) and stayed elevated until postexercise (+24%, *p* < 0.001), but returned to baseline at 24 h (−15%, *p* = 0.003 to postexercise). Neither TIMP-1 concentrations (Table 1) nor MMP activity (Figure 2A) significantly changed throughout the study.

**TABLE 1:** Parameters of endothelial matrix remodeling.

	**Blood withdrawals**			
**Parameters**	**Baseline**	**Arrival**	**Postexercise**	**24 h**
MMP-9 (ng/ml)	585.2 (330.1)	315.9(121.8)^†^	140.7(119.2)^†^	404.9(198.0)*
MMP-2 (ng/ml)	185.4 (27.2)	217.0(25.0)^††^	229.7(25.8)^†⁣††^	196.4(32.8)**
TIMP-1 (ng/ml)	149.8 (22.1)	161.4 (19.6)	155.9 (34.7)	172.5 (24.3)
SDF-1 (pg/ml)	295.4 (191.6)	425.9 (239.2)	249.0 (109.4)	216.7 (96.0)
IL-3 (pg/ml)	56.4 (36.2)	127.2(61.9)^†^	121.9(61.2)^†^	86.7 (42.3)

Data are given as mean (standard deviation). *n* = 10, except IL-3 *n* = 7; baseline: 519 m a.s.l.; arrival: hypobaric hypoxia after passive ascent to an altitude of 3,883 m a.s.l.; postexercise: after 2 h of acute hiking exercise (±400 m elevation gain); 24 h: after an overnight stay at 3,883 m a.s.l. Significant differences to baseline and to the preceding time point are indicated as follows: ^†^*p* < 0.05; ^†⁣†^*p* < 0.01; ^†⁣†⁣†^*p* < 0.001, and * *p* < 0.05; ** *p* < 0.01, respectively. MMP, matrix metalloproteinase; TIMP-1, tissue inhibitor of MMP-1; SDF-1, stromal cell-derived factor-1; IL-3, interleukin-3.

### Parameters of Progenitor Cell Mobilization

Stromal cell-derived factor-1 did not significantly change during the study (Table 1). IL-3 significantly increased at arrival (+126%, *p* = 0.022), stayed there until postexercise (+116%, *p* = 0.028), and returned to baseline at 24 h (Table 1).

### Cell and Serum or Plasma Parameter Correlations

Endothelial cell concentrations were significantly associated with MMP-9 concentrations (*r*_rm_ = 0.48, *p* = 0.010, *n* = 9, Figure 2B), but not with MMP activity. Neither CPC nor EC numbers were significantly related to SDF-1α or IL-3 concentrations.

### Leukocyte Counts

An overview of leukocyte counts is given in Table 2. Total white blood cells significantly increased postexercise (+32%, *p* = 0.002 to arrival; +65%, *p* = 0.011 to baseline) and decreased back to baseline the next morning (−42%, *p* = 0.004). Absolute and relative monocytes did not significantly change throughout the study. Absolute neutrophils significantly increased postexercise (+97%, *p* = 0.034 to baseline) and stayed there until 24 h (+60%, *p* = 0.045 to baseline). Absolute and relative lymphocytes were unchanged at arrival and postexercise compared to baseline and decreased at 24 h (absolute counts: −42%, *p* = 0.005 to postexercise; relative counts: −32%, *p* = 0.040 to baseline). Relative neutrophils showed a biphasic increase above baseline (+25%, *p* = 0.016 and +33%, *p* = 0.026 at arrival and 24 h).

**TABLE 2:** Overview leukocyte counts.

**Leukocyte counts**	**Blood withdrawals**			
	**Baseline**	**Arrival**	**Postexercise**	**24 h**
WBC (10^9^/l)	5.57 (2.72)	6.96 (2.33)	9.16(2.16)^†,^**	5.31(0.55)**
Lymphocytes (10^3^/μl)	1.95 (0.69)	2.01 (0.59)	2.49 (0.60)	1.45(0.40)**
Monocytes (10^3^/μl)	0.53 (0.34)	0.40 (0.19)	0.52 (0.11)	0.36 (0.24)
Neutrophils (10^3^/μl)	3.05 (1.84)	4.70 (1.51)	6.01(1.99)^†^	4.86(2.48)^†^
Lymphocytes (%)	35.40 (9.57)	27.65 (4.56)	27.41 (7.46)	23.97(9.87)^†^
Monocytes (%)	9.15 (5.42)	5.58 (2.84)	5.56 (0.94)	5.61 (3.33)
Neutrophils (%)	50.36 (8.60)	62.70(4.35)^†^	62.84 (8.48)	67.06(13.74)^†^

Data are given as mean (standard deviation). *n* = 10; baseline: 519 m a.s.l.; arrival: hypobaric hypoxia after passive ascent to an altitude of 3,883 m a.s.l.; postexercise: after 2 h of acute hiking exercise (± 400 m elevation gain); 24 h: after an overnight stay at 3,883 m a.s.l. Significant differences to baseline and to the preceding time point are indicated as follows: ^†^*p* ≤ 0.05 and ** *p* < 0.01, respectively. WBC, white blood cell count.

## Discussion

This is the first study to report that acute exercise at high altitude limits EC shedding in unacclimatized subjects on a group level, while an acute exercise bout in hypobaric hypoxia did not increase regenerative CPC concentrations as expected. Results were not linked to endothelial matrix remodeling or CPC mobilization.

Although exercise for 30 min under normobaric hypoxia enhanced EC shedding (Tsai et al., 2016), the authors reported a reduced increase in ECs after a preconditioning to hypoxic stress by high-intensity interval training (Chen et al., 2015). Unexpectedly, our results on average even showed an EC decrease after 2 h of acute hiking at high altitude. These results extend existing literature (Mancuso et al., 2008), where ECs were shown to decline after a multiple-day trek at high altitude. The authors suggested hypoxia-induced angiogenesis and an enhancement of ECs in the pulmonary circulation priming the vasculature for long-term structural adaptations (Howell et al., 2003) as an underlying cause. This effect is unlikely to have happened short term. Importantly, in our study, the attenuation of endothelial shedding was not linked to MMP activity and therefore endothelial matrix remodeling, but unexpectedly to a reduction in circulating MMP-9 protein concentrations. This could be due to most of MMP-9 being released from circulating ECs or even point toward a reduced secretory function of monocytes and macrophages under hypobaric hypoxia as shown *in vitro* (Rahat et al., 2006). Apart from ECs, leukocytes are known to accumulate MMP-9 in their secretory granules, so they are the most likely source of circulating MMP-9 concentrations. Gelatinase levels in systemic blood reflect the balance between their release into and their removal from the bloodstream. As such—although these two processes are not yet well understood—gelatinase measurement in serum can be useful to detect the systemic response to acute exercise (Lo Presti et al., 2017). Furthermore, the loss of circulating MMP-9 concentrations could also implicate an attenuation of macrophage infiltration and collagen deposition in circulation (Luttun et al., 2004), but was not found to be any indicator of an exercise-induced change in muscular damage (Lo Presti et al., 2017).

Circulating MMP-2 concentrations, however, were not associated with EC number. As such, the increase in circulating MMP-2 concentrations until postexercise seen in our study could simply be a direct consequence of acute exercise under hypoxic conditions as a proangiogenic stimulus in muscular tissue (Chen et al., 2013).

It is important to note that acute exercise at high altitude showed a different systemic effect on the circulating protein levels of MMP-9 and MMP-2 as well as their inhibitor TIMP-1, which suggests that these proteins might have very different time courses of expression/secretion/activity. Under normoxic conditions, MMP-9 concentrations immediately increased after acute exercise, while MMP-2 and TIMP-1 concentrations either increased or stayed unchanged (Lo Presti et al., 2017). A decrease in MMP-9 concentrations was only observed under hypoxic conditions without exercise stimulus (Rahat et al., 2006). Although adhesion of leukocytes to VCAM-1 expressed by the endothelium upon transendothelial migration evoked a rapid transcriptional response manifested in the synthesis of only MMP-2 messenger RNA (mRNA) within 30 min (no MMP-9 mRNA expression) and protein translation, MMP enzyme activity could not be detected until 12 h after adhesion (Yakubenko et al., 2000). These different time courses of protein expression/secretion/activity could be one reason why only MMP-2 concentrations were upregulated until postexercise in our study and MMP activity did not significantly change on a group level throughout the study duration.

Analysis of the individual responses of endothelial shedding to acute exercise at high altitude, however, showed that subjects experiencing a cell decline postexercise (56%) were not necessarily those showing an increased EC concentration at arrival (22%). Responders that encountered a hypoxia-induced increase in endothelial shedding most certainly also experienced an exercise-induced EC decrease at high altitude. However, there were also non-responders to the hypoxic stimulus experiencing the same exercise-induced EC effect. This could be due to genetic differences between subjects, which would affect gene induction/repression—possibly regulated via microRNAs—in a low oxygen environment (Fasanaro et al., 2008). Individual differences in single nucleotide polymorphisms could also influence MMP expression/activity under hypoxic conditions (Franczak et al., 2019).

Absolute EC changes from arrival to postexercise were positively associated with the relative exercise dose, possibly implying less limitation of EC shedding with more strenuous exercise at high altitude. Here, it is important to note that subjects did not walk as a group at a similar absolute exercise intensity, but at their individual pace as fast as possible, which resulted in a specific exercise dose (intensity, duration) for each subject—represented by the time-weighted average HR as percent predicted of HR_max_. However, differences between subjects could also result from different fitness levels (fitter individuals have a lower HR at the same pace), and changes in HR also occur with altitude. Therefore, a certain acclimatization effect cannot be excluded.

Unlike exhausting exercise in normobaric hypoxia (Kroepfl et al., 2012), the acute exercise bout at high altitude on average could not enhance regenerative CPC number. However, absolute CPC change from arrival to postexercise was positively related to the individual relative exercise dose, meaning the higher on average the relative exercise dose, the less CPC reduction induced at high altitude. Although normobaric hypoxia in healthy subjects at rest in two different studies showed opposing CPC changes (Ciulla et al., 2007; Colombo et al., 2012), our data are more in line with the study by Colombo et al. (2012)—possibly because Ciulla et al. (2007) analyzed cells of a more mature phenotype. The authors’ explanation for hypoxia-induced CPC reduction was an increase in CXCR-4 on the cells’ surface helping them to quickly respond to a high expression of SDF-1α in peripheral tissues. Exercise-induced CPC concentrations at high altitude in our study were neither related to SDF-1α nor to IL-3 plasma concentrations, which would argue against the exercise-induced push–pull mechanism—local factors within the bone marrow niche “push” CPCs to the circulation, while systemic factors in blood “pull” CPCs from the bone marrow (Emmons et al., 2016). It is rather likely that CPC migration was triggered by tissue hypoxia, as shown after a 12-day trek at high altitude (Mancuso et al., 2008). Our findings extend these results and show that an acute short-term exercise bout at high altitude could not increase regenerative CPC concentrations on a group level, although the absolute CPC change was exercise-dose dependent. Therefore, a certain exercise-induced CPC mobilization at high altitude is likely, and possibly mediated by oxidative stress (Kroepfl et al., 2012) through an adrenergic mechanism (Kropfl et al., 2014).

### Limitations

Owing to the field character of the study, subjects spent their free time between postexercise and 24 h doing some unsupervised recreational activities. The lack of a non-exercising control group at altitude is a limitation. Therefore, it is impossible to exactly state what effects are attributed to the altitude and what effects are attributable to the bout of exercise at altitude. However, an isolated exercise effect should have been detected based on already observed EC (Tsai et al., 2016) and CPC changes (Colombo et al., 2012; Kroepfl et al., 2012) and the outcome of the *a priori* power analysis. In addition, the study design could have benefited from a timed control that did not reside at altitude to control for diurnal cell variation. Although the lack of standardization of the exercise bout and no repeated measures at different exercise intensities within the same individual limit the study design, cell changes from arrival to postexercise were significantly related to individual HR data as percent predicted of HR_max_.

## Conclusion

A single acute exercise bout at high altitude on average attenuated endothelial shedding, but could not increase regenerative CPC concentrations. The exercise-induced limited EC response in hypobaric hypoxia was not linked to endothelial matrix remodeling by MMP activity. The lack of an exercise-induced CPC increase at high altitude was not attributable to the SDF-1α or IL-3 trafficking pathways. However, individual responses to hypoxia alone and acute exercise at high altitude showed responders and non-responders for EC changes as well as MMP-activity—possibly due to genetic differences between subjects. This study provides information to better understand the changes in the endothelium and the immature immune system during an active, short-term sojourn at high altitude at a group and an individual level. In addition, cell changes during the acute exercise bout at high altitude were dependent on the relative exercise dose, which is important information for planning any exercise intervention in these extreme environments. Future studies should focus on differentiating between the hypoxia- and exercise-induced effects as well as the influence of an exercise dose manipulation at high altitude on cell concentrations. In doing so, one could ideally elucidate the specific exercise dose that would limit endothelial shedding, but at the same time counteract the decline of regenerative immature immune cells at high altitude.

## Data Availability Statement

All datasets generated for this study are included in the article/supplementary material.

## Ethics Statement

The studies involving human participants were reviewed and approved by the Local Ethics Committee of the University of Munich, Germany (Project No. 350-16). The patients/participants provided their written informed consent to participate in this study.

## Author Contributions

JK, TK, MR, IS, SS, and CS contributed to the conception and design of the work. JK, TK, VF, JS, H-JG, SS, and CS were involved in the acquisition, analysis, and interpretation of the data for the work. All authors drafted the work or revised it critically for important intellectual content, approved the final version of the manuscript, and agreed to be accountable for all aspects of the work in ensuring that questions related to the accuracy or integrity of any part of the work were appropriately investigated and resolved. All persons designated as authors qualify for authorship, and all those who qualify for authorship are listed.

## Conflict of Interest

The authors declare that the research was conducted in the absence of any commercial or financial relationships that could be construed as a potential conflict of interest.
